# Acute severe idiopathic lymphoid interstitial pneumonia: A case report

**DOI:** 10.1097/MD.0000000000021473

**Published:** 2020-07-24

**Authors:** Youssef Lamkouan, Sandra Dury, Jeanne Marie Perotin, Remi Picot, Anne Durlach, Olivier Passouant, Sebastian Sandu, Maxime Dewolf, Antoine Dumazet, François Lebargy, Gaëtan Deslee, Claire Launois

**Affiliations:** a Department of Pulmonary Medicine; b Equipe d’Accueil 4683; c Institut National de la Santé Et de la Recherche Médicale Unité Mixte de Recherche en Santé 1250, University of Reims Champagne-Ardenne; d Hippocrate Laboratory; e Department of Biopathology; f Department of Intensive Care Medicine; g Department of Thopracic Surgery, University Hospital of Reims, Reims, France.

**Keywords:** interstitial lung disease, lymphoproliferative, lymphoid, rituximab

## Abstract

**Rationale:**

Lymphoid interstitial pneumonia is a rare benign pulmonary lymphoproliferative disorder usually presenting with a sub-acute or chronic condition and frequently associated with autoimmune disorders, dysgammaglobulinemia, or infections.

**Patient concerns:**

A 74-year-old woman with no past medical history presented with acute dyspnea, nonproductive cough, hypoxemia (room air PaO_2_: 48 mmHg) and bilateral alveolar infiltrates with pleural effusion. Antibiotics and diuretics treatments did not induce any improvement. No underlying condition including cardiac insufficiency, autoimmune diseases, immunodeficiency, or infections was found after an extensive evaluation. Bronchoalveolar lavage revealed a lymphocytosis (60%) with negative microbiological findings. High-dose intravenous corticosteroids induced a mild clinical improvement only, which led to perform a surgical lung biopsy revealing a lymphoid interstitial pneumonia with no sign of lymphoma or malignancies.

**Diagnoses:**

Acute severe idiopathic lymphoid interstitial pneumonia.

**Interventions:**

Ten days after the surgical lung biopsy, the patient experienced a dramatic worsening leading to invasive mechanical ventilation. Antibiotics and a new course of high-dose intravenous corticosteroids did not induce any improvement, leading to the use of rituximab which was associated with a dramatic clinical and radiological improvement allowing weaning from mechanical ventilation after 10 days.

**Outcomes:**

Despite the initial response to rituximab, the patient exhibited poor general state and subsequent progressive worsening of respiratory symptoms leading to consider symptomatic palliative treatments. The patient died 4 months after the diagnosis of lymphoid interstitial pneumonia.

**Lessons:**

Idiopathic lymphoid interstitial pneumonia may present as an acute severe respiratory insufficiency with a potential transient response to rituximab.

## 1 Introduction

Lymphoid interstitial pneumonia (LIP) was first described by Liebow and Carrington in the late 1960s as a benign lymphoproliferative disorder limited to the lungs and characterized by diffuse infiltration of the alveolar septa by dense collections of polyclonal lymphocytes associated with plasma cells and other cellular elements.^[1–5]^ LIP is usually associated with systemic immunological disorders including autoimmune diseases, dysglobulinemia, and infections. The 2 main conditions associated with LIP are Sjögren syndrome for autoimmune diseases and human immunodeficiency virus (HIV) for infections.^[3–5]^ Among other conditions, systemic lupus erythematosus, rheumatoid arthritis, myasthenia, Hashimoto thyroiditis, autoimmune hemolytic anemia, allogenic bone marrow transplantation, pulmonary alveolar microlithiasis, pulmonary alveolar proteinosis, common variable immune deficiency, diphenylhydantoin use, and various infections including Epstein-Barr virus (EBV), Human T-cell lymphotropic virus-1 (HTLV-1), *Legionella*, tuberculosis, *Mycoplasma*, and *Chlamydia* have also been reported to be associated with LIP.^[3–5]^ Idiopathic LIP is rare with limited available information regarding its clinical/radiological features and prognosis.^[3–6]^ The clinical presentation of LIP is classically characterized by an insidious onset with exertional dyspnea and nonproductive cough, and in some cases associated with general symptoms including fever, night sweats, and weight loss.^[6–8]^

We describe herein an unusual case of acute severe idiopathic LIP with a transient response to rituximab.

## 2 Case report

**Figure 1:**
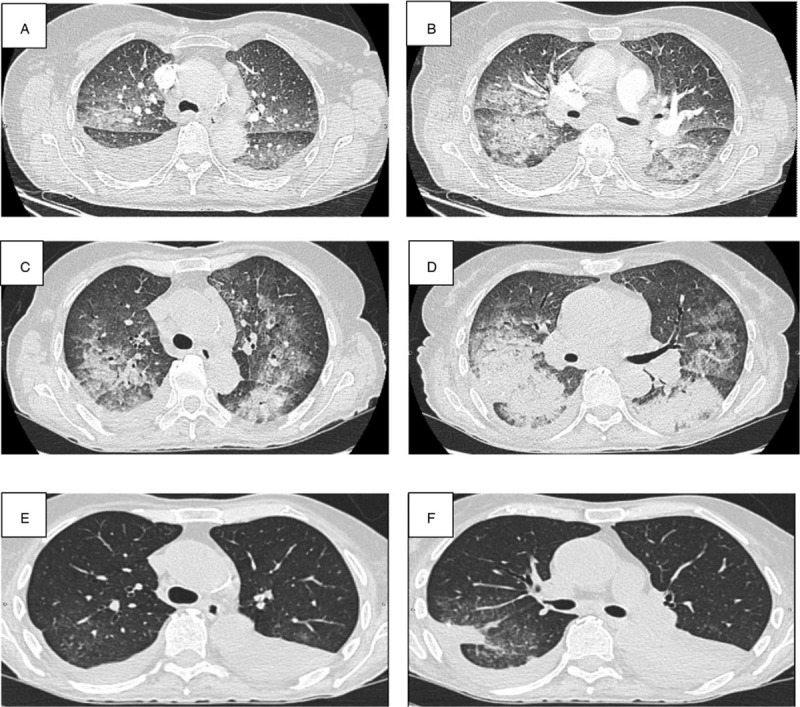
High resolution CT. Chest CT-scan at the initial presentation (A, B), after exacerbation (C, D), and after rituximab treatment (E, F). CT = computed tomography

**Figure 2:**
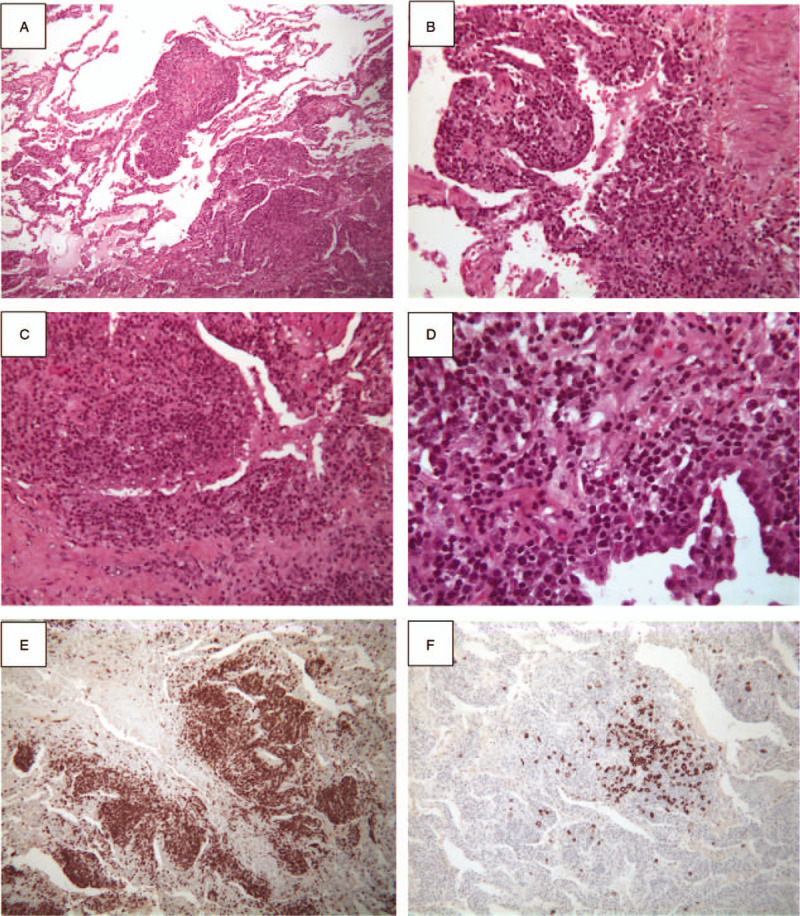
Histopathology of the surgical lung biopsy. HES stain shows a dense interstitial lymphoid proliferation involving alveolar walls over the large areas of the lung with some nodular infiltration in some areas (A, B, C, D, respectively ×25, ×50, ×100, and ×250). Lymphocytes show positive stain for T-cell marker (CD3) (E, ×25) whereas some lymphocytes are positive for B-cell marker (CD20) (F, ×25). HES = hematoxylin-eosin-saffron.

A 74-year-old woman without any medical history was admitted for progressive worsening of dyspnea and nonproductive cough without fever for 4 weeks. Arterial blood gas revealed severe hypoxemia (room air PaO_2_: 48 mm Hg) and hypocapnia (PaCO_2_: 29 mm Hg). Chest computed tomography (CT)-scan revealed bilateral alveolar infiltrates with no cyst, pleural effusion, and no sign of pulmonary embolism (Fig. 1A and B). Cardiac echography did not find any sign of cardiac insufficiency. No improvement was obtained after antibiotics and diuretics treatments. A bronchoalveolar lavage was performed showing 73 × 10^3^ cells per mL with 60% lymphocytes and 40% macrophages with no specific cytologic or microbiological findings. No autoimmune disease was identified with no clinical sign of extrathoracic manifestation and no specific biological findings including antinuclear antibody 1/400, negative Sjögren syndrome-related antigen A (anti-Ro) and B (anti-La), negative anti-cyclic citrullinated peptide antibody, negative rheumatoid factor, normal serum electrophoresis, no immunoglobulin deficiency, normal thyroid function, and negative EBV, HIV, and HTLV-1 serologies. Pulmonary function tests revealed a low diffusing capacity (DL_CO_: 48%) with no obstructive or restrictive pattern. Intravenous corticosteroids were started (250 mg/d for 3 days) and then 1 mg/kg oral, leading to a mild clinical improvement. Because of uncertain diagnosis, a surgical lung biopsy was proposed. At this step, the main diagnosis hypotheses were carcinomatous lymphangitis, hematolymphoid malignancies including lymphoma, and idiopathic LIP. Lung biopsy analyses revealed a typical aspect of LIP with no sign of malignancies or lymphoma (Fig. 2). The lung parenchyma was involved by dense and interstitial lymphoid proliferation localized in alveolar walls over large areas of the lung. The lymphocytes were essentially CD3+ and only few CD20+ without tumoral pattern or cellular atypia. Plasma cells demonstrated a polyclonal pattern of expression for kappa and lambda light chains. Because of the rarity of LIP and the unusual clinical and radiological presentation, pathology was assessed by 2 independent teams confirming the diagnosis of LIP, ruling out differential diagnoses of lymphoma, and other lymphoproliferative disorders including IgG4-related disease and Castelman disease.

Ten days after the surgical lung biopsy, the patient presented clinical and radiological worsening (Fig. 1C and D) with acute respiratory distress leading to admission to Intensive Care Unit. No improvement was obtained after treatment with Optiflow Nasal High Flow, antibiotics, diuretics, and a new course of intravenous corticosteroids (500 mg/d for 3 days and then 1 mg/kg), requiring invasive mechanical ventilation. After a multidisciplinary discussion, rituximab was started (1000 mg on both day 1 and day 15) leading to a dramatic clinical and radiological improvement (Fig. 1E and F) allowing weaning from mechanical ventilation 10 days after day 1 of rituximab treatment. The patient state subsequently improved allowing to stop oxygen therapy after 4 weeks. Despite the initial response to rituximab, the patient exhibited poor general state and progressive worsening of respiratory symptoms leading to consider symptomatic palliative treatments. The patients died 4 months after the diagnosis of LIP obtained by surgical lung biopsy.

According to the Jardé law in France, informed consent for inclusion was waived based on the retrospective non-interventional design and anonymous management of the patients’ data and was approved by the French national commission for personal data protection (CNIL, Comité National de l’Information et des Libertés) (n°2049775 v 0).

## 3 Discussion

We present herein an unusual case of acute severe idiopathic LIP necessitating invasive mechanical ventilation with a transitory response to rituximab.

One of the unusual aspect of this idiopathic LIP is its acute clinical course with symptoms starting 4 weeks before hospitalization, and presenting with an acute respiratory insufficiency subsequently necessitating invasive mechanical ventilation. The onset of LIP is usually insidious with symptoms duration before diagnosis ranging from 2 months to 12 years and typically exceeding 3 years.^[3,6]^ To our knowledge, no other case of LIP presenting as an acute respiratory insufficiency necessitating invasive mechanical ventilation has been described before. However, we cannot rule out that the surgical lung biopsy may have been involved in the exacerbation of LIP, as reported in other interstitial lung diseases.^[9]^

Despite an extensive evaluation, no underlying disease was found in this case leading to classify this LIP as idiopathic. It must be pointed out that idiopathic LIP is a rare condition. In a series of 15 cases of LIP, Cha et al^[6]^ found only 3 cases of idiopathic LIP, and no idiopathic LIP was found in a series of 13 LIP described by Strimlan et al.^[8]^ Of notes, idiopathic LIP is reported mainly in men, whereas our patient was a woman. However, despite an extensive evaluation which did not identify an underlying condition, we cannot completely rule out that an underlying disease which was not present at the time of the LIP diagnosis may have appeared overtime. Moreover, a malignant transformation of LIP to lymphoma has also been described,^[10]^ which may have occurred overtime.

Another unusual feature of this case is the absence of cysts, whereas chest CT-scan shows cysts in 80% of patients with LIP.^[3]^ Cysts have typically thin-walled and random distribution with a size usually <30 mm. Cysts are particularly frequent when associated with underlying Sjögren syndrome.^[3]^ Pleural effusion and consolidation as described in our case are rare in LIP,^[3,4]^ and these features are considered as signs of malignancies including lymphoma. In this clinical case, the diagnoses of lymphoma or other malignancies were carefully ruled out by 2 independent pathologists who clearly described a polyclonal pattern of the lymphocytic infiltration. No other lymphoproliferative disorders including IgG4-related disease and Castelman disease were found.

Finally, one of the main finding of this clinical case is the transitory response to rituximab, whereas corticosteroids had not induced significant clinical and radiological improvement. In idiopathic LIP, corticosteroids usually exhibit a good response with stabilization or improvement in 50% to 60% of the cases.^[4,6]^ The use of other immunosuppressive agents including hydoxychloroquine, azathioprine, cyclosporine A, and mycophenolate have also been described with limited data regarding the safety and efficacy of these treatments.^[3]^ Rituximab use has been reported in one case of LIP associated with common variable immune deficiency^[11]^ with a sustained complete remission. In our patient, rituximab also induced a dramatic clinical and radiological improvement. However, this effect was transient with subsequent poor general state and worsening of clinical symptoms leading to death.

In conclusion, this case highlights that idiopathic LIP may present as an acute severe respiratory insufficiency, and that rituximab might be associated with a favorable clinical and radiological response. However, it should be pointed out that the response to rituximab was transient in this case, and that additional data are clearly needed before considering rituximab as a validated therapeutic option in LIP.

## Author contributions

All the authors have been involved in conceptualization, investigation, resources, visualization, writing – original draft, and writing – review and editing.
